# Creation of Sexually Transmitted Diseases Education Program for Young Adults in Rural Cambodia

**DOI:** 10.3389/fpubh.2019.00050

**Published:** 2019-03-14

**Authors:** Joseph Kai Man Kam, Lok Ki Wong, Kirsten Ching Wah Fu

**Affiliations:** ^1^Faculty of Medicine, School of Public Health and Primary Care, The Chinese University of Hong Kong, Shatin, Hong Kong; ^2^Stanley Ho Centre for Emerging Infectious Diseases, Postgraduate Education Centre, Prince of Wales Hospital, The Chinese University of Hong Kong, Shatin, Hong Kong; ^3^Buddies for Life, Hong Kong, Hong Kong

**Keywords:** sexually transmitted diseases, health education, intervention, rural population, Cambodia

## Abstract

**Introduction:** Globally, adolescents and young adults aged from 15 to 24 years accounted for 25% of the sexually active population. They also took up almost 50% of all newly acquired sexually transmitted diseases (STDs) worldwide. In developing countries/ regions, there have been more unreported cases due to the limited resources and availability of data. This project aimed to enhance sexual health knowledge and encourage behavioral change in sexual practice in rural Cambodia by educational interventions.

**Methods:** A multi-prong project used the Theory of Planned Behavior (TPB) as framework to guide the implementation, which involved carrying out educational talks, group discussion sessions. The educational health talks described the signs and symptoms of STDs and preventive measures including the Abstinence-Be faithful- Condom (ABC) strategy. Pre- and post-intervention assessments were conducted on 111 villagers aged between 18 and 30 years who lived in Peaksneng Thyme Village, Cambodia. Special materials were prepared that highlighted such information were also given to all participants. This was followed by discussion sessions that aimed to strengthen an appropriate attitude toward STDs. A pilot trial was done to prepare and build a constructive, realistic atmosphere and facilitated in-depth spread of main messages. A 20-item assessment of STD knowledge was used as pre- and post-intervention evaluation tool. Individual participant scores were compared to determine the effectiveness of interventions.

**Conclusion:** This comprehensive program was effective in enhancing sexual knowledge in high-risk groups of both sexes as well as general public in rural Cambodia. With experiences gained, this could be replicated in nearby communities, possibly motivating community behavioral change in future. Alternatively, this approach could be further developed, or using other behavioral theories, and applied to other health issues in this country.

## Introduction

The global adolescent population has risen to the highest level in recent history. This population (15 to 24 years old) is responsible for 25% of those who are sexually active. Yet they represent almost 50% of all newly acquired sexually transmitted diseases (STDs) worldwide (1).

Cambodia is one of the world's poorest nations. In 2017, its total Gross Domestic Product (GDP nominal) was $22 billion, yielding a per capita GDP of just $1400, among the lowest in the world. It is also one of the rapidly developing countries in Southeast Asia, has also one of the youngest populations in the region. In their last population census, two-thirds of its 14 million citizens were aged 25 years or younger, and one fifth were young adults aged between 15 and 24 years (2). The proportion of women in the adult population is high, 56% of those who are 18 years old or more being females. Also, as a result of the war, there is a rather high proportion of women-headed household; at least 25% according to UNICEF. (Official Royal Government of Cambodia Website (English Version): https://www.mef.gov.kh/documents/shares/CMM_Mid-2016-Assessment-English-Version.pdf; accessed February 12th, 2019).

Among the top ten categories of “unpleasant” diseases in Cambodia, STDs ranked first amongst males and second for females (1). These figures reflected the high prevalence of various STDs in the local community. According to the current UNAIDS Global Report, the estimated human immunodeficiency virus (HIV) infection prevalence amongst adults aged 15 to 49 years in Cambodia was 0.6%, while the AIDS-related deaths among them was 2000 in 2015 (3). For syphilis and gonorrhea, the estimated prevalence in adults over 15 years were 4 and 3%, respectively since 1990s (4). These showed that STDs are of epidemic scale, presenting enormous health and economic burden for the country.

A brief literature review showed a plethora of commentaries and researches that emerged on this issue, although data are still limited regarding actual incidence/ prevalence of STDs in rural areas of Cambodia due to geographical and political restraints. A cross-sectional survey conducted in 2003 attempted to describe the sexual behavior of 665 unmarried men aged 15 to 24 years in the marginal areas of Phnom Penh. It showed that 39% of the respondents had given money or gifts in exchange for sex (5). Another local survey in the same year found that 42% of rural sexual workers were HIV positive; 22% had past or current syphilis (6). These findings revealed a potentially high burden of STDs among residents in rural Cambodia. This cultural transition in sexual norms and practice were experienced by the population of this developing country. In particular, the low literacy level and associated paucity in sexual knowledge among rural residents gave rise to risky behavior including unprotected sex with casual partners, and conferred a greater risk for transmission of STDs (7, 8). Behavioral patterns and prevention needs of this distinct population, therefore, deserved focused attention (9, 10). Educational, health and social programs must promptly reach them with necessary information, skills, services and supplies that they crucially need in order to attain their own protection from unwanted or unsafe sexual practice (11). This gave the basis for motivating the creation of educational program of STDs in Peaksneng Thyme Village in rural Cambodia.

The main goal was to reduce vulnerability of villagers to STDs, particularly young adults through educational, health and social programs. Taking account of ethical consideration, only villagers aged 18 years or above were recruited. The primary goal was to achieve a minimum 30% improvement in the post-test after the program was implemented. They were also expected to have a better basic understanding of STDs through participation in the program. These included knowledge of basic clinical signs and symptoms, as well as the means for prevention of these diseases. Moreover, this program aimed at pioneering an educational concept that appropriate sexual knowledge should be bred from adolescence in daily lives. It acted as the first step to arouse public awareness of different health concerns in the Peaksneng Commune.

## Materials and Methods

### Setting

The creation and implementation of program was at the Peaksneng Thyme Village, situated northwest of Siem Reap, Cambodia, with a rural community of around 2000 population. Half of the villagers were aged below 18 years and 75% of them dropped out from high school. The target study population were young adults, from 18 to 29 years old due to the ethical concern. The sample size initially aimed at around 100 participants and ended up with 111 participants recruited from convenient sampling method of available households/ individuals at time of study by the village representative. More females participated because that was busy time for farmers; and many males have to work in the farm fields, thus not available. The program was fully backed up by a non-government charity organization (NGO) named Buddies for Life from Hong Kong which collaborated during the whole creation process. This charity had been organizing a children center in the village for past two years, and already gained trustworthiness as well as ability to solicit supportive actions from the locals. It facilitated this project by not only providing a suitable site, but also arranging the logistics and language translators for different activities. It also helped with the liaison, in securing sample size and guarding quality of data. In November 2017, a site visit was done and a pilot group of participants was formed. The official program was finalized in late-January 2018. Written informed consents were distributed to participants to address possible ethical concerns. A pre-test assessment of participants was then completed for baseline evaluation, followed by a health talk and question-and-answer (Q&A) sessions at community level for knowledge consolidation. Post-test assessment was performed using the same set of questions right afterwards (around 30 min) to evaluate the outcomes, specifically the effectiveness of project.

### Framework

In order to attain a reasonable and comprehensive program, the Theory of Planned Behavior (TPB) was used as framework to guide the implementation (12). This was seen to be particularly effective for developing primary preventive interventions. It assumes human behavior to be rational and led by logical thinking, highly predictive of human functioning, and served as a model for empirical studies of behavioral prediction and change. This theory states that human behavior is usually guided by three kinds of considerations, viz: behavioral beliefs, normative beliefs and control beliefs. In their respective aggregates, behavioral beliefs produce either a favorable or unfavorable “attitude toward the behavior”, while normative beliefs result in “subjective norm” and control beliefs give rise to “perceived behavioral control”. In combination, these three constructs lead to the formation of a “behavioral intention.” In particular, “perceived behavioral control” is presumed to not only affect actual behavior directly, but also affect it indirectly through behavioral intention (13). As a general rule, the more favorable the attitude toward behavior and subjective norm, and the greater the perceived behavioral control, the stronger the person's intention to perform the behavior in question should be. Finally, given a sufficient degree of actual control over the behavior, people are expected to carry out their intentions when the opportunity arises (14). By utilizing the observed statistical relationships between internal constructs based on *behavioral, normative, and controlled beliefs* for retrospective analysis, predictive investigations and design of interventions could be achieved (14). Taking sexual behavior as working example, we made use of TPB to ameliorate the mistaken practices and perceptions. The three constructs of TPB have previously been proven to be useful in HIV prevention research in predicting both the intention and the actual use of condoms by clients in diverse settings (15). Therefore, a questionnaire comprising four categories (Knowledge of STDs, Attitude toward STDs, Subjective norms and Perceived behavioral control to sex) was designed for pre- and post-intervention analysis. With an appropriate application of TPB in designing the pre- and post-tests and the accompanying health education talks, a better understanding of the villagers' intention and practices could be obtained. The program could then adopt a more targeted and validated approach, hence resulting in their sexual behavior to be more likely adjustable afterwards.

### Methodology

#### Pilot Group

The Peaksneng Thyme Village was one of six villages in the Peaksneng Commune. There is no physical boundary but only administrative difference amongst them. A single primary road was recognizable while houses were built in scattered fashion. Villagers traveled around basically on foot or by bicycles. A classroom inside the primary school, built with bricks, and cements, was chosen as the site of health talks.

In November 2017, plans were finalized for preparation of layout and implementation of the program. An in-depth site visit was led by the village representatives. Meanwhile, a pilot group of 10 participant villagers was formed after consensus was reached by discussion with the village representatives, as well as the organizers of the study. These were informal meetings which enabled good communications of intentions and plans of work. They agreed not only to discuss sensitive topics with the interviewer, but promised to participate in the later parts of program as well. Importantly, this provided critical baseline information for further assessment, in particular, of villagers who did not have previous exposures to any STD education.

Baseline information included population size, land area, basic infrastructure (water, electricity), accessibility of the population, general education level, and time duration that could be acceptable to participate in the study. A general background of STDs in Peaksneng was subsequently obtained by verbal interviews with this small pilot group through the assistance of translators. They conveyed their personal/ family experiences of STDs as well as how they approached prevention and control measures with traditional beliefs and practices. A productive interaction resulted despite some embarrassment during the process.

A few uncontrollable factors occurred which led to ultimate partial completion of survey in this pilot group, including limitation of time which was worse than expected as villagers focused on their farm work during the harvesting season. In addition, some personal interviews were interrupted by bad weather. In spite of these difficulties, rapport and trustworthiness were concretely built with the community through an effective communication process. On the other hand, the pilot trial enabled the translators to a longer preparation time, thus providing for a more accurate interpretation on unusual vocabularies used in the pre- and post-tests. It also ensured the consistency of translation during the health talks later.

#### Pre- and Post-tests

In January 2018, convenient sampling was started to include 111 participants by the village representative. These included both males and females aged from 18 to 29 years, at the project site in the Peaksneng Thyme Village. Written informed consents were obtained from each of them at the beginning of program. This was followed by anonymous questionnaires for collection of demographic data.

A set of 20 questions covering knowledge, behavioral, normative and controlled beliefs of STDs were designed as contents of the pre- and post-intervention assessments. Indeed, a number of questions were gone through beforehand in order to select appropriate ones to be integrated. The finalized questionnaire was constructed mainly according to the authors' own field experience. Since the primary objective of the program was to enhance the basic understanding of STDs, in particular knowledge among the participants, a relatively large proportion of questions focused on the facts concerning STDs.

As a result, seven questions (including Q1, Q6, Q7, Q8, Q15, Q16, Q17, and Q19) were extracted from the category that pertained to participants' knowledge of STDs. For the remaining questions, an appropriate application of the TPB theory was applied. Four questions (including Q2, Q9, Q13, and Q18) concerned the participants' attitude toward STDs. Five questions (including Q3, Q4, Q5, Q12, and Q20) were related to the subjective norms while three questions (including Q10, Q11, and Q14) corresponded to participants perceived behavioral control toward sexual behavior.

The written tests were delivered in English language, and the questions were asked sequentially in English one-by-one. After Q1 was raised, for example, it was repeated immediately in Khmer by the two interpreters (one male and one female, for respective participants of same gender). The interpreters lived in the village and possessed English proficiency at university level). Participants followed the instructions and wrote down their own answers. The interpreters ensured every participant catch up with the answering pace before moving on to the next question.

Extra buffering time was allowed because of possible language barrier and the whole process took more than 1 h to complete for one participant. With the assistance of two interpreters (one male and one female, for respective participants of same gender, living in the village with fluent English at university level) from the NGO, most of the participants were able to complete the tests individually under mass translation (one interpreter speaking to a group of participants) during both pre- and post-intervention test periods. We considered this gender- gender approach is important for the participants, as this respected their local culture and helped to gain rapport between the interpreters and participants. Moreover, when the discussions were organized, the participants already had enough confidence that their questions/ ideas could be effectively communicated.

#### Health Education Sessions

In accordance with traditional Cambodians customs, two sessions of health talks were designed by the authors separately for demonstrating basic STDs knowledge to male and female participants. The teaching materials available from the Department of Health in Hong Kong were the primary reference resources. (https://www.chp.gov.hk/en/healthtopics/content/24/1607.html, accessed October 20th 2018).

Each educational session lasted for 3 h, including the completion of the pre- and post-intervention tests. HIV/AIDS, the major global health problem reported by the WHO, was illustrated as a core example of typical STDs. Other STDs including gonorrhea, chlamydia and bacterial vaginosis were also briefly mentioned. Simple identifications of common physical signs and symptoms, as well as use of appropriate preventive measures were foci of this phase of health communication.

A regular genital self-examination was encouraged to look for any abnormalities including sores, discharge and other problems such as genital warts. Since there was success of prevention strategies that addressed primary sexual behavior by adopting the ABC strategy in Uganda (9), this method was also briefly introduced. The approach stands for “Abstinence, Be faithful, use a Condom”. It emphasizes the effectiveness of a safe sex on minimizing the spread of STDs by combining the risks avoidance and harm reduction. Its positive impact has further been confirmed by a subsequent study (16). Thus, crucial concepts of “using abstinence until marriage,” “eliminating casual or other concurrent sexual relationships,” and “using condom for protected sex” had been explained in details to the participants.

During the education process, there were opportunities to move beyond providing health information to further address the importance of health issues, other communication and health decision-making situations. Both females and males enjoyed an equality of opportunity to receive education and rewards in the program. They were also welcomed to ask and share their opinions all along with the help of translators.

Both printed and electronic media were then used to deliver key messages. Nevertheless, the effectiveness of reading materials was at times not fully commendable due to the low educational level of many rural villagers. An interactive Q&A session was arranged to overcome the constraints of mass illiteracy. Strong and supportive feedbacks were then observed among the participants. Some sensitive questions were actively raised. For instance, one of the female participants showed her concern on distinguishing the usual discharge during pregnancy from the signs and symptoms of STDs. There were a lot of subsequent discussions, with provision of some gifts and snacks as incentives. All participants received a gift-set comprising of a T-shirt, a pencil bag with a sharpened pencil, and a pack of biscuits or instant noodles at the end of the program.

#### Statistical Calculations

The IBM SPSS Statistics version 23 software was used to analyze distributions and calculate significance in comparisons between groups. McNemar's test was used to further analyze the corresponding significance of individual question in the pre- and post- intervention assessments. The mean score was derived based on the number of correct responses for all of the test questions. Subgroup analyses of each category were performed, as well as overall comparison, of pre- and post- test improvements by summing up the number of correct responses for all items in each construct. A two-sided *p*-value of <0.05 was taken as indicative of significance.

#### Ethics Statement

This study was carried out in accordance with the recommendations of Ethical Guidelines for Human research Involving Human Subjects, Ministry of Health, Kingdom of Cambodia. The whole program has been approved by the Survey and Behavioral Research Ethics Committee of the Chinese University of Hong Kong. All subjects gave written informed consent in accordance with the Declaration of Helsinki.

## Results

### Descriptive Analysis

Among the 111 participants included in the program, the proportion of males and females were 38.7 and 61.3%, respectively (Table 1). The median age was 25 years including some who had received schooling, with illiteracy rate of 19.8% (22/111). More than half were currently under education or work for living. Slightly more than half (59.5%) of respondents were married, while almost three-quarters (71.9%) claimed to be sexually-experienced. Majority of them (82%) had also heard about STDs before joining the program.

**Table 1:** Demographic characteristics of study population.

**Characteristic (*N* = 111)**	***N* (%)**
**SEX**	
Female	68 (61.3%)
Male	43 (38.7%)
**AGE (YEARS)**	
18 to <19	22 (19.8%)
19 to <20	2 (1.8%)
20 to <21	6 (5.4%)
21 to <22	8 (7.2%)
22 to <23	2 (1.8%)
23 to <24	5 (4.5%)
24 to <25	9 (8.1%)
25 to <26	23 (20.7%)
26 to <27	12 (10.8%)
27 to <28	10 (9.0%)
28 to <29	2 (1.8%)
29 to <30	4 (3.6%)
≥ 30	6 (5.4%)
Median age	25
**EDUCATION**	
Primary	28 (25.2%)
Secondary	59 (53.2%)
University	2 (1.8%)
*Illiterate*	22 (19.8%)
**OCCUPATION**	
Student	23 (20.7%)
Worker	12 (10.8%)
Farmer	23 (20.7%)
Housewife	49 (44.1%)
Miscellaneous	4 (3.6%)
**MARITAL STATUS**	
Single	45 (40.5%)
Married	66 (59.5%)
Widowed	0 (0%)
**SEXUAL EXPERIENCE**	
Yes	79 (71.2%)
No	32 (28.8%)
**EVER HEARD ABOUT STDS**	
Yes	91 (82%)
No	20 (18%)

### Changes in Degree of Knowledge

Analysis and comparison between the pre- and post-intervention tests showed that the mean score, derived based on the number of correct responses for all of the test questions, of pre-test was 7.7 out of 20 with a right-skewed distribution. The post-test score became 12.8 out of 20, likely in a normal distribution. This represented more than 60% improvement when comparing the mean scores of the pre- and post-intervention tests. Also, the overall correct responses, from 111 participants, improved from total of 855 pre-test to 1,421 post-test.

In general, the results showed significant differences and that participants, through the health education process, had a better factual knowledge of STDs and relevance to their sexual practices in daily lives.

By using the paired *t*-test for further analysis, *p* < 0.001 could be obtained which implied a statistically significant positive effect of the health intervention. We were able to show that the program was beneficial to the participants' degree of knowledge at time of pre- and post-tests. Although there were differences in the levels of improvement in each question, all except question 13 showed at least 30% of progression in the post-test (Tables 2a,b).

**Table 2a:** Categories that pertained to knowledge of STDs in relation to participant's gender.

	***N* = number of participants who obtained correct answer in Pre-test (Sex)**	***N* = number of participants who obtained correct answer in Post- test (Sex)**	***p*- value (McNemar Test)**
**KNOWLEDGE OF STDS**			
1) HIV/AIDS is an STD.	40 (M) 46 (F)	43 (M) 68 (F)	0.25 (M) <0.001 (F)^*^
6) Painful sex is a possible symptom of an STD.	9 (M) 28 (F)	27 (M) 30 (F)	<0.001 (M)^*^ 0.5 (F)
7) Painful urination is a possible symptom of an STD.	19 (M) 29 (F)	32 (M) 30 (F)	<0.001 (M)^*^ 1.000 (F)
8) Having discharge from vaginal/penis is a symptom of an STD.	17 (M) 18 (F)	25 (M) 25 (F)	0.008 (M) 0.016 (F)
15) STDs left untreated can cause deafness and death in their later stages.	22 (M) 31 (F)	33 (M) 48 (F)	0.001 (M)^*^ <0.001 (F)^*^
16) STDs can destroy a person's ability to have children.	17 (M) 30 (F)	23 (M) 55 (F)	0.07 (M) <0.001 (F)^*^
17) STDs can be passed on from mother to child.	16 (M) 29 (F)	26 (M) 50 (F)	0.002 (M)^*^ <0.001 (F)^*^
19) There are vaccines (such as gardasil) for STDs preventions.	9 (M) 22 (F)	34 (M) 38 (F)	<0.001 (M)^*^ <0.001 (F)^*^
Knowledge total	149 (M) 233 (F)	243 (M) 344 (F)	<0.001 (M)^*^ <0.001 (M)^*^

* p ≤ 0.05 (two sided): significant difference between pre- and post- tests scores.

*M, male; F, female*.

**Table 2b:** Categories that pertained to attitude, subjective norms and perceived behavioral control, in relation to participant's gender.

	***N* = obtaining correct answer in Pre-test (Sex)**	***N* = obtaining correct answer in Post-test (Sex)**	***p*-value**
**ATTITUDE TO STDS**			
2) Condoms give a full protection from AIDS.	8 (M) 23 (F)	33 (M) 54 (F)	<0.001 (M)^*^ <0.001 (F)^*^
9) You are more likely to get STDs if being sexually active with more than one person.	14 (M) 21 (F)	28 (M) 22 (F)	<0.001 (M)^*^ 1.000 (F)^*^
13) Being tested for an STD can be painful and embarrassing.	14 (M) 35 (F)	24 (M) 36 (F)	0.002 (M) 1.000 (F)
18) You will contract STDs if you are young.	17 (M) 32 (F)	29 (M) 39 (F)	0.001 (M)^*^ 0.016 (F)^*^
Subtotal	53 (M) 111 (F)	114 (M) 151 (F)	<0.001 (M)^*^ <0.001 (F)^*^
**SUBJECTIVE NORMS**			
3) Early-stage STDs without symptoms are contagious.	15 (M) 22 (F)	25 (M) 57 (F)	0.002 (M)^*^ <0.001 (F)^*^
4) STDs show symptoms straight away.	18 (M) 29 (F)	30 (M) 57 (F)	0.002 (M)^*^ <0.001 (F)^*^
5) You can have an STD without symptoms.	11 (M) 25 (F)	24 (M) 52 (F)	<0.001 (M)^*^ <0.001 (F)^*^
12) You can recover from STDs with receiving treatment.	13 (M) 22 (F)	28 (M) 24 (F)	<0.001 (M)^*^ 0.5 (F)
20) STDs are unpreventable and incurable.	15 (M) 24 (F)	36 (M) 45 (F)	<0.001 (M)^*^ <0.001 (F)^*^
Subtotal	72 (M) 122 (F)	143 (M) 235 (F)	<0.001 (M) <0.001 (F)
**PERCEIVED BEHAVIORAL CONTROL TO SEX**			
10) If you have any symptoms of STD, you should visit doctor and avoid having sex.	24 (M) 19 (F)	30 (M) 35 (F)	0.07 (M) <0.001 (F)^*^
11) Abstinence helps prevent the spread of STDs.	12 (M) 22 (F)	24 (M) 31 (F)	<0.001 (M)^*^ 0.004 (F)^*^
14) When the symptoms go away, it means the STD is cured.	9 (M) 29 (F)	31 (M) 40 (F)	<0.001 (M)^*^ 0.001 (F)^*^
Subtotal	45 (M) 70 (F)	85 (M) 106 (F)	<0.001 (M)^*^ <0.001 (F)^*^

* p ≤ 0.05 (two sided): significant difference between pre- and post- tests scores.

*M, male; F, female*.

### Gender Effect

McNemar's test was used to further analyze the corresponding significance of individual question in relation to possible gender differences, with statistically different results (*p* ≤ 0.05) obtained for both sexes in: Q15, 19 (knowledge); 2, 9 (attitude); 4, 5 (subjective norms). However, only males showed significant improvements in Q6, 7 (knowledge); 18 (attitude); 12 (subjective norms); 11, 14 (perceived behavioral control), while the corresponding categories found only amongst females were Q 1, 16, 17; 3, and 10. These gender differences might imply cultural practices in their upbringing or socialization process. In particular, women and men have different and special needs, that would need be addressed separately, as well as common ones across groups.

Amongst the eight questions related to the categories of knowledge of STDs (Table 2a), question 8 showed the lowest rate of accuracy (*N* = 50). This was likely explainable by the problem of understanding by the participants. For example, the technical term “discharge” stated in the question was hard to comprehend within such a short time. In contrast, “painful urination” as a recognizable subjective symptom was comprehended well amongst men (*p* < 0.001) who showed significant improvement which was apparently not seen in women.

On the other hand, it is notable that more than half of the participants failed to get correct answers of question 9, 11, and 12 separately (Table 2b). These questions were related to all three constructs of the Theory of Planned Behavior, which meant attitude, subjective norms and perceived behavioral control. This possibly implied that the effectiveness of the program, in terms of achieving successful application of TBP, might be doubtful.

### Literacy Effect

Although the baseline characteristics showed that participants might be restricted by their educational level, apparent significant advancement was gained in the overall outcome assessment. Tables 3a,b shows the effects of literacy analyzed pertaining to different categories. Overall improvements could be seen for both literate and illiterate groups of participants (*p* < 0.001). On further examination, however, it can be seen that while the literate (*N* = 89) showed improvements in 80.0% (16/ 20) questions, the illiterate (*N* = 22) could only show significant improvements for 25.0% (5/ 20), in questions 1, 2, 3, 4, 5. This highlights the importance of fundamental language education in communicating the messages of the program. Subgroup analysis of the illiterate group showed that all questions showed improvement except those pertaining to “perceived behavioral control to sex” which did not reach significant (*p* = 0.077) difference between pre- and post- tests. This will be an important target area in future improvements of the program.

**Table 3a:** Categories that pertained to knowledge of STDs in relation to participant literacy.

	***N* = number of participants who obtained correct answer in Pre-test (literacy)**	***N* = number of participants who obtained correct answer in Post-test (literacy)**	***p*- value (McNemar Test)**
**KNOWLEDGE OF STDS**			
1) HIV/AIDS is an STD.	84 (L) 2 (I)	89 (L) 22 (I)	0.063 (L) <0.001 (I)^*^
6) Painful sex is a possible symptom of an STD.	30 (L) 7 (I)	49 (L) (I)	<0.001(L)^*^ 1.000(I)
7) Painful urination is a possible symptom of an STD.	38(L) 10 (I)	51 (L) 11 (I)	<0.001 (L)^*^ 1.000 (I)
8) Having discharge from vaginal/penis is a symptom of an STD.	31 (L) 4 (I)	46 (L) 4 (I)	<0.001 (L)^*^ 1.000 (I)
15) STDs left untreated can cause deafness and death in their later stages.	60 (L) 10 (I)	69 (L) 12 (I)	<0.001 (L) 0.5 (I)
16) STDs can destroy a person's ability to have children.	43 (L) 9 (I)	61(L) 17(I)	<0.001 (L)^*^ 0.008 (I)^*^
17) STDs can be passed on from mother to child.	38 (L) 13 (I)	60(L) 16(I)	<0.001(L)^*^ 0.250 (I)
19) There are vaccines (such as gardasil) for STDs preventions.	37 (L) 8 (I)	61 (L) 11 (I)	<0.001 (L)^*^ 0.25 (I)
Knowledge subtotal	361 (L) 63 (I)	486 (L) 101 (I)	<0.001 (L)^*^ <0.001 (I)^*^

* p ≤ 0.05 (two sided): significant difference between pre- and post- tests scores.

*L, literate; I, illiterate*.

**Table 3b:** Categories that pertained to attitude, subjective norms and perceived behavioral control, in relation to participant literacy.

	***N* = obtaining correct answer in Pre-test (literacy)**	***N* = obtaining correct answer in Post-test (literacy)**	***p*-value (McNemar Test)**
**ATTITUDE TO STDS**			
2) Condoms give a full protection from AIDS.	25 (L) 6 (I)	68 (L) 19 (I)	<0.001 (L)^*^ <0.001 (I)^*^
9) You are more likely to get STDs if being sexually active with more than one persons.	27 (L) 8 (I)	42 (L) (I)	<0.001(L)^*^ 1.000(I)
13) Being tested for an STD can be painful and embarrassing.	30 (L) 10 (I)	49 (L) 11 (I)	0.002 (L)^*^ 1.000 (I)
18) You will contract STDs if you are young.	32 (L) 12 (I)	53 (L) 15 (I)	<0.001 (L)^*^ 0.25 (I)
Subtotal	114 (L) 36 (I)	212 (L) 53 (I)	<0.001 (L)^*^ <0.001 (I)^*^
**SUBJECTIVE NORMS**			
3) Early-stage STDs without symptoms are contagious.	33 (L) 4 (I)	63 (L) 19 (I)	<0.001 (L)^*^ <0.001 (I)^*^
4) STDs show symptoms straight away.	39 (L) 8 (I)	67 (L) 20 (I)	<0.001 (L)^*^ 0.001 (I)^*^
5) You can have an STD without symptoms.	28 (L) 8 (I)	59 (L) 17 (I)	<0.001 (L)^*^ 0.004 (I)^*^
12) You can recover from STDs with receiving treatment.	30 (L) 5 (I)	46 (L) (I)	<0.001 (L)^*^ 1.000 (I)
20) STDs are unpreventable and incurable.	66 (L) 10 (I)	67 (L) 14 (I)	1.000 (L) 0.125 (I)
Subtotal	196 (L) 35 (I)	302 (L) 76 (I)	<0.001 (L)^*^ <0.001 (I)^*^
**PERCEIVED BEHAVIORAL CONTROL TO SEX**			
10) If you have any symptoms of STD, you should visit doctor and avoid having sex.	37 (L) 6 (I)	56 (L) 9 (I)	<0.001 (L)^*^ 0.25 (I)
11) Abstinence helps prevent the spread of STDs.	27 (L) 7 (I)	44 (L) (I)	<0.001(L)^*^ 0.125 (I)
14) When the symptoms go away, it means the STD is cured.	39 (L) 9 (I)	58 (L) 13 (I)	<0.001 (L)^*^ 0.125 (I)
Subtotal	103 (L) 22 (I)	158 (L) 33 (I)	<0.001 (L)^*^ 0.016 (I)^*^

* p ≤ 0.05 (two sided): significant difference between pre- and post- tests scores.

*L, literate; I, illiterate*.

For the process evaluation, some checking questions (questions 3, 4, and 5) were included to assess possible language constraint. In real practice, for instance, there was no such word in Khmer that bear an equivalent meaning of “contagious.” This exposed a risk of miscomprehension after translation of text to local language. The questions then functioned as a tool to counter-check the consistency of participants' responses. In particular, the pre-test in the pilot group provided essential feedbacks from participants for fine adjustments.

A few planned activities like communications platform with the local health-care providers and set-up of a condom distribution center had not been carried out. Although the completeness of those early data was limited due to the sudden interruption due to bad weather, the acceptability of respondents toward STDs could still be observed and assessed. In addition, core families (which had the closest relationships with the interpreters) were selected for more in-depth interactions at subsequent educational sessions. These demonstrations in families thus subsequently influenced others in the community to encourage a higher level of concentration during the health communication. Participants felt less shy but more comfortable throughout the program. There was no embarrassment as expected in the process of sexual education. These ensured the overall feasibility of the program in this part of the country.

During the outcome evaluation, the pre- and post-intervention questionnaires with the prior instruments provided good evidence that high proportion of participants fulfilled the learning objectives.

## Discussion

### Strengths

This creation was one of the pioneering programs of sexual education in the Peaksneng Commune, in rural Cambodia. There were similar studies and previous projects related to this topic in Cambodia; yet none of them covered the same region as the Peaksneng Thyme Village, which is far away from Phnom Penh. Though there were significant researches in much larger scale previously (17, 18), some of the results might be questionable. For instance, according to the report released by the World Health Organization (WHO) in 2001, there was huge success in the implementation of the 100% Condom Use Program in Cambodia (19). Their data collection, however, depended on self-reporting from official disciplines and brothel workers. Those figures might be biased and not able to mirror the reality. Our present program in Peaksneng Thyme Village complemented the data coverage for rural parts of the population, despite its relatively small scale. We demonstrated notable gender disparities in different questions which can be good indicators for future development of program. We also showed that although literacy is an important hurdle for health education, particular risk areas could be effective communicated with enough tools (including good translation) in place.

### Limitations

There are several limitations of this study project. Firstly, at the organizational level, this program took place precisely at the first stage of developing a sustainable education in Peaksneng Thyme Village (as planned and supported by NGO). We envisage that through development in future, there should be better indicators for outcome evaluation rather than purely relying on the results of pre- and post-intervention tests. These can include behavioral changes relating to sexual practices, as relates to abstinence and partner faithfulness in particular. Secondly, the program design was solely based on the TPB and the interventions were completed using this approach. We saw many inadequacies in this study with regards to TPB, viz: there are issues with items used to assess the Attitude, Subjective Norm (SN), and Perceived Behavioral Control (PBC) measures, as these measures did not seem to assess respective constructs. Instead, these seem more suitable to measure a mix of knowledge, vulnerability to getting STDs (e.g., Q 9 and 18), response efficacy (e.g., Q 12), severity (e.g., Q 20) and response cost (e.g., Q 13). These constructs seem to fit better with other concepts like Protection Motivation Theory instead of TPB.

Thirdly, the coverage of questions did not include whether participants had any intention to alter their sexual behavior. Specifically, we did not measure proof of behavioral change after the program, at this time point of project. There is also the question of how illiterate participants could be better reached. While we used printed materials and books were given as reference materials for the community, these would not be useful for the illiterate people.

Noteworthy are some key points in the questionnaire, such as the need to visit the doctor and abstain from sex was only endorsed by 58% of the participants. This absolutely key point might not have been emphasized enough during the sessions. Some of the statements were also problematic and might cause problems in interpreting responses. For example, Q15 seemed to have implication that all STDs cause deafness and death which is incorrect. Statement 20 is in fact two statements—STDs are unpreventable and STDs are incurable. This might be difficult to know which of these two points the participant is focusing on and there is no reason to believe their response would be identical to both aspects of the statement.

At the societal level, sexual issues were an existing taboo in traditional customs in Cambodia. Rural residents particularly women were even more sensitive to this topic. Having a low social and economic status, they were traditionally seen as the “giver” or “creator” of STDs. A previous study in 2,000 reported that, within their cultural norms, they were to be discouraged from being knowledgeable about sexual matters through peer pressure (10). Such gender bias easily led to inequalities like dependence on males and the opportunity of receiving sexual education. It has been found that many Cambodian women are heavily reliant upon the opinions of their husbands, communities and elders when deciding on family planning (20).

Although females were successfully recruited for participation in this project, their cultural behavior could not seemingly be influenced at all (21). One reason was likely because of the short-term nature of present program. It seemed certain that there must be more obstacles for promoting a sustainable education on STDs in future (22, 23). Specifically, the high drop-off rate from the program was a definite concern. The reasons behind could be the absence of personal interests on the issues, or the forbiddance of attending activities outside traditional family values in the community.

In addition, from the individual point of view, miscommunication caused by dialect was another pitfall. This highlighted the importance of quality translators who were crucial in programs. Double translation had been avoided to minimum by collaborating well with the interpreters from the Peaksneng Commune. However, it is foreseeable that the degree of difficulty might increase gradually along with deepening level of knowledge. The interpreters were lay persons who had not been professionally trained in healthcare matters. They might not be familiar with the theoretical terms. Future programs could possibly mitigate the language barrier, for instance, by training the local interpreters prior to the program.

A limitation of our study was that the participants lost their concentration easily during the program. It is understandable that the language barrier hindered their interest in learning, though incentives could be given by providing gifts and rewards. The problem of limited time frame for interventions remained unsolved. This was critical in that individual participant response might wax and wane with a longer span of time. The program may then not have the same sustainable significance in a more lengthy duration.

In spite of the thorough preparation with the NGO beforehand, there were many unexpected challenges in real practice. Some planned activities like basic screening and referral services were inapplicable as these were deemed impracticable at that time. Not only man-power and resources were insufficient, but time was inadequate as well as the planned agenda was moving much slower than expected. It was also harvesting season for villagers in November to March, so they could be available for only a few hours per day. There was very limited time on personal counseling. Despite some embarrassment, most participants exhibited great passion, kindness and interests in the program. There were surprising interactions with them, making the event more fulfilling for both parties. These led to the modifications and elimination of some interventions during time of execution.

The Cambodian government's advocacy of natural family planning also favored the program to a large extent. Some messages concerning sexual behavior have been spread, guided by the official government. It then became reasonable that an atmosphere of change in cultural acceptance, at least through this kind of discussion, had been formed. While 12.5% of Cambodian women had still unmet family planning needs (18), they appeared much willing to acquire more in-depth information regardless of the traditions. In their opinion, they typically related natural family planning to the issue of sex education. This seemed to correlate well with the basic tenet that sex education was the pillar of STDs prevention. It was probably also because of this reason that the program did arouse their interests and thus gained support from more than 100 participants in the Peaksneng Commune. This changing culture may thus be construed as constructive to sustainable health education.

## Conclusion

To conclude, there is a continuing need to educate rural population in Cambodia on the risk for acquiring STDs and their formidable sequelae; and to increase the importance of abstinence, faithfulness and, when these fails, use of barrier methods in sex. This project emphasized the importance of sustainable education on sexual behavior, particularly for rural areas with low accessibility and literacy rates. The STDs education program for adolescents and young adults in Peaksneng Thyme Village had merits in knowledge distribution and inspiring behavioral change. As it is an ongoing development as planned with the NGO, the outcome of possibly effective behavioral change could not yet be measured. However, results up to the present evaluation of the STD education program strongly pointed to be affirmative. Sustainable efforts would need be made in the program development. Hopefully, a more concrete and measurable change in their sexual behavior could be seen in near future.

## Author Contributions

JK was responsible for the conception of this study, checked and analyzed data, and wrote the final version of this manuscript. LW is the main data contributor of this project. KF initiated and coordinated the field work in Cambodia.

### Conflict of Interest Statement

The authors declare that the research was conducted in the absence of any commercial or financial relationships that could be construed as a potential conflict of interest.
